# Computationally Efficient *p*-Version Finite Element Analysis of Composite-Reinforced Thin-Walled Cylindrical Shells with Circumferential Cracks

**DOI:** 10.3390/ma18071404

**Published:** 2025-03-21

**Authors:** Jae S. Ahn

**Affiliations:** School of General Education, Yeungnam University, Gyeongsan 38541, Republic of Korea; jsahn@ynu.ac.kr

**Keywords:** thin-walled cylindrical shells, circumferential cracks, composite patch, *p*-version finite element method, stress intensity factor

## Abstract

Cylindrical shells are extensively employed in fluid transport, pressure vessels, and aerospace structures, where they endure mechanical and environmental stresses. However, under high pressure or external loading, circumferential cracks may develop, threatening structural integrity. Composite patch reinforcement is an effective method to mitigate crack propagation and restore structural performance. This study presents a finite element model using *p*-refinement techniques to analyze cylindrical shells with circumferential cracks reinforced by composite patches. The approach integrates equivalent single-layer (ESL) and layer-wise (LW) theories within a unified single-element mesh, significantly reducing the degrees of freedom compared to conventional LW models. Fracture analysis is conducted using the virtual crack closure technique (VCCT) to evaluate stress intensity factors. The model’s accuracy and efficiency are verified through benchmark and patch reinforcement simulations. Additionally, a parametric study examines how patch material, thickness, and adhesive properties affect reinforcement efficiency across varying crack angles. This study provides an effective methodology for analyzing composite-reinforced thin-walled cylindrical shells, offering valuable insights for aerospace, marine, and pipeline engineering.

## 1. Introduction

Cylindrical shells are extensively utilized in fluid transport systems, high-pressure enclosures, and aerospace applications due to their strength and capacity to withstand internal pressure. While axial cracks are commonly associated with internal pressure, high-pressure environments induce substantial tensile stress in the pipe wall, making circumferential cracks a critical failure mode [1]. Additionally, external factors such as bending, deformation, and impact loads, along with environmental conditions like ground settlement and corrosion, further accelerate crack propagation. These factors collectively contribute to structural degradation, highlighting the need for effective repair and prevention strategies to ensure long-term reliability and safety. Among various reinforcement techniques, composite patch reinforcement has emerged as a promising solution due to its effectiveness in mitigating circumferential cracks.

Composite patch reinforcement has been widely adopted as an effective method for restoring structural integrity and inhibiting crack propagation, particularly in cylindrical shells, as it offers an efficient and lightweight solution with a high strength-to-weight ratio and excellent fatigue resistance [2]. Composite patches provide a cost-effective alternative to conventional metal reinforcements while delivering superior mechanical performance. Since its initial development in the late 1970s, composite patch reinforcement has evolved beyond its early applications on flat plates, where it was primarily used to enhance durability and slow crack propagation [3,4,5,6,7,8,9,10]. With growing interest in the reinforcement of cylindrical structures, particularly pipelines, recent research has focused on developing improved techniques to address crack mitigation under complex loading conditions. Achour et al. [11] demonstrated the effectiveness of bonded composite wraps in reinforcing cracked pipes subjected to bending loads, while Rashed et al. [12] analyzed the influence of patch thickness and placement on crack propagation in circumferentially cracked pipes. Budhe et al. [13] investigated the failure pressure of composite-repaired pipelines, emphasizing the impact of plastic deformation on structural integrity. Localized composite patching has also been explored as a repair method for through-wall cracks, with Jamal-Omidi et al. [14] confirming its effectiveness in restoring mechanical performance. Yu et al. [15] evaluated composite repair techniques for large-diameter pipes with severe metal loss, and Savari [16] examined composite patch reinforcement in spiral-welded pipes, demonstrating its role in crack mitigation and reliability improvement. Various numerical modeling approaches, particularly finite element methods (FEMs), have been applied to optimize these repair techniques. Advanced meshing strategies, including hexahedral and tetrahedral elements for three-dimensional simulations and shell elements for two-dimensional modeling, were employed to enhance accuracy [17,18,19,20].

In recent decades, various modeling approaches have been developed to enhance the accuracy and efficiency of composite structure analysis [21]. Among these, equivalent single-layer (ESL) theory and layer-wise (LW) theory are particularly relevant for composite patch repairs. ESL theory, which has evolved primarily from classical plate theory and first-order shear deformation theory, is now implemented in commercial finite element software such as ANSYS 2024 R2 and ABAQUS 2024. This approach provides reliable predictions for the global behavior of thin laminated composite structures, including displacements, critical buckling loads, and fundamental vibration frequencies [22]. However, ESL theory assumes continuous in-plane displacement, making it less effective in capturing local stress variations in thick laminates, particularly the zigzag displacement patterns commonly observed in laminated composites [23,24,25].

To address these limitations and accurately model micro- and macro-scale damage mechanisms, such as matrix cracks, interlaminar delamination, debonding, fiber breakage, and transverse cracks, LW theory provides a more refined approach [26]. Unlike three-dimensional theories, LW theory does not directly use three-dimensional shape functions but instead combines two- and one-dimensional shape functions [22]. This method improves computational efficiency by differentiating the relatively small thickness direction using one-dimensional shape functions while employing two-dimensional plane subdivision [27]. However, although more efficient than fully three-dimensional models, LW theory significantly increases the degrees of freedom (DOF) compared to ESL-based elements, thereby reducing computational performance [26].

The *p*-refinement FEM improves solution accuracy by increasing the polynomial order of shape functions instead of refining the mesh by adding more elements, as in conventional *h*-refinement FEM. This approach is particularly beneficial for analyzing structures with cracks and stress singularities, as it allows for the precise capture of localized stress distributions [27]. Furthermore, *p*-refinement FEM enhances computational efficiency by achieving faster convergence with fewer elements, thereby reducing modeling complexity and computational costs. Due to these advantages, it has been extensively studied and applied in various engineering disciplines, particularly in fracture mechanics and structural analysis [28,29,30,31]. A previous study [28] proposed transition elements utilizing the *p*-version FEM to efficiently analyze patch repairs for cracked plates. These elements connect ESL plate elements in the global region with LW plate elements in the local region. In another study [29], *p*-version LW shell elements were used to analyze cylindrical shells with cracks repaired using composite patches. When using transition elements, the analysis typically requires integrating ESL elements, LW elements, and transition elements, increasing the complexity of the modeling effort. On the other hand, while using only LW elements simplifies the modeling process and ensures high accuracy, it significantly increases the number of DOF, leading to higher computational costs and reduced efficiency.

This study presents an enhanced finite element formulation that integrates the advantages of ESL and LW theories into a unified single-element mesh. By eliminating the need for transition elements, this approach significantly reduces computational complexity and DOF while maintaining high numerical efficiency and accuracy. Fracture analysis is conducted using the virtual crack closure technique (VCCT), based on linear elastic fracture mechanics, to evaluate the stress intensity factors. The proposed model is verified through benchmark studies on orthotropic laminated shell elements and then applied to thin-walled cylindrical shells with circumferential cracks reinforced by composite patches. Additionally, a parametric study examines the influence of key factors such as patch material, thickness, and adhesive properties across varying crack angles to assess their effect on reinforcement efficiency. The findings offer valuable insights into improving composite patch reinforcement strategies for aerospace, marine, and pipeline engineering applications.

## 2. Materials and Methods

### 2.1. Construction of Polynomical Shape Functions for p-Version FEM

In the *p*-version FEM, shape functions are commonly represented using either Lagrange or Legendre polynomials [32,33]. Shape functions based on Legendre polynomials provide excellent numerical stability due to their orthogonality. However, Legendre polynomials themselves do not inherently ensure continuity at element boundaries, which is essential for accurate numerical solutions. To address this limitation, Lobatto shape functions, which are derived from the integral form of Legendre polynomials, are widely used. A key advantage of Lobatto shape functions is their ability to ensure continuity at element boundaries. While Legendre polynomials offer orthogonality, they do not guarantee smooth transitions between adjacent elements, leading to potential discontinuities in the numerical solution. In contrast, Lobatto shape functions are designed to satisfy continuity conditions at the element boundaries, making them particularly suitable for FEM applications. Additionally, Lobatto shape functions contribute to improved numerical stability, particularly in high-order approximations. When the polynomial order of the shape function increases, shape functions based solely on Legendre polynomials can exhibit oscillatory behavior within the elements, potentially reducing numerical accuracy. However, Lobatto shape functions, by imposing constraints at the element boundaries, reduce such oscillations and provide a more stable numerical representation [34]. This study utilizes Lobatto shape functions, which are derived from Legendre polynomials to ensure continuity between finite elements. The shape function variables are classified into nodal and modal components. Nodal variables correspond to specific geometric locations and carry physical meaning, whereas modal variables are independent of geometric positioning and primarily refine the accuracy of nodal values. Unlike nodal variables, modal variables do not possess direct physical significance but contribute to improved numerical precision. The one-dimensional Lobatto shape functions *A* for any given *p*-level are formulated as follows:

(1) $$\begin{array}{l} A_{0}(x)=\frac{1-x}{2},\;\;A_{1}(x)=\frac{1+x}{2}\\ A_{i}(x)=\sqrt{\frac{2i-1}{2}}\int_{-1}^{x}\frac{1}{2^{s-1}(s-1)!}\frac{d^{i-1}}{ds^{i-1}}(s^{2}-1{)}^{i-1}ds,\;\;\;\;\;i=2,\;\;3,\;\;4,\ldots ,p \end{array}$$

In the *xy* plane, two-dimensional shape functions are constructed based on one-dimensional shape functions. These two-dimensional functions consist of nodal modes and modal shapes. Specifically, the nodal modes are derived by combining two one-dimensional shape functions.

(2) $$B_{1+i+3j-2ij}(x,y)=A_{i}(x)A_{j}(y)\;\mathrm{in}\;i,j\;=\;0,1$$

The nodal modes *B* correspond to the linear Lagrange interpolation in two dimensions. The modal shapes *C* are categorized into side shapes and internal shapes. Equation (3) presents the side shapes for any given *p*-level, where the superscripts in the equation indicate side numbers.

(3) $$\begin{array}{l} C_{i}^{1}(x,y)=A_{0}(y)A_{i}(x)\\ C_{i}^{2}(x,y)=A_{1}(x)A_{i}(y)\\ C_{i}^{3}(x,y)=A_{1}(y)A_{i}(x)\\ C_{i}^{4}(x,y)=A_{0}(x)A_{i}(y) \end{array}\;\mathrm{with}\;2\;\leq \;i\;\leq \;p$$

The internal shapes are given by Equation (4).

(4) $$C_{i}^{5}(x,y)=A_{s}(x)\;A_{t}(y)\;\mathrm{in}\;s\;=\;2,\;3,\ldots ,\;p\;-\;2\;\mathrm{and}\;t\;=\;p\;-\;s;\;i\;=\;1,\;2,\ldots (p\;-\;3)(p\;-\;2)/2$$

### 2.2. Geometry and Displacement Fields

In a cylindrical coordinate system, nodal modes are preferred over a general Cartesian system for linear mapping of geometric fields. Unlike *p*-refinement, where higher-order modes are introduced, the number of nodal modes remains unchanged, allowing for a simplified mesh structure. A three-dimensional geometric configuration in cylindrical coordinates is derived from one-dimensional nodal modes *A*_0_ and *A*_1_, combined with the two-dimensional nodal modes *B*.

The displacement fields, including in-plane components *u^c^* (*c* = 1, 2) and the out-of-plane component *u*^3^, are expressed using Lobatto shape functions as follows:

(5) $$\begin{array}{l} u^{c}(x,\theta ,r)\;=B_{i}(x,\theta )\;\left[A_{0}(r)\;\alpha_{i1}^{c}+\;A_{1}(r)\alpha_{i2}^{c}\right]\;\;+\;C_{j}^{s}(x,y)\left[A_{0}(r)\;\beta_{j1}^{c}+\;A_{1}(r)\;\beta_{j2}^{c}\right]\\ u^{3}(x,\theta )\;=B_{i}(x,\theta )\alpha_{i1}^{3}\;\;\;+\;C_{j}^{s}(x,\theta )\beta_{j1}^{3}\;\;\;\;\;\;\;\;\;\;\;\;\;i=1,\;2,\;3,\;4\;;\;\;\;j=1,\;2,\;\ldots ,\;n\;\;;\;\;\;s\;\;=\;\;1,\;2,\;3,\;4,\;5 \end{array}$$

Here, the indices *i*, *j*, and *s* adhere to the Einstein summation convention. The parameter *n* indicates the number of variables along each of the two-dimensional plane for *s* = 1, 2, 3, and 4, while for *s* = 5, it corresponds to the number of internal variables within the two-dimensional domain. The nodal variables are denoted as *α*, whereas the modal variables are represented by *β*. The shape functions *A*_0_, *A*_1_, and *B* act as linear basis functions, with *A*_0_ and *A*_1_ being one-dimensional and *B* being two-dimensional. Additionally, *C* serves as two-dimensional shape functions for modal variables, constructed from a combination of one-dimensional Lobatto shape functions *A*.

### 2.3. Constitutive Equations

Under the plane stress assumption, the strain components {*ε*} in the cylindrical coordinate system (*x*,*θ*,*r*) can be simplified from three-dimensional theory of elasticity, as shown in Equation (6).

(6) $$\begin{array}{l} \{\varepsilon \}\;=\;<\;\varepsilon_{x}\;\;\;\;\varepsilon_{\theta }\;\;\;\;\gamma_{x\theta }\;\;\;\gamma_{\theta r}\;\;\;\gamma_{rx}{>}^{T}\\ \;\;\;\;\;\;\;=<\;\frac{\partial u^{1}}{\partial x}\;\;\;\;\;\frac{u^{3}}{r}+\frac{1}{r}\frac{\partial u^{2}}{\partial \theta }\;\;\;\;\;\frac{1}{r}\frac{\partial u^{1}}{\partial y}+\frac{\partial u^{2}}{\partial x}\;\;\;\;\;\;\frac{1}{r}\frac{\partial u^{3}}{\partial \theta }+\frac{\partial u^{2}}{\partial r}-\frac{u^{2}}{r}\;\;\;\;\;\frac{\partial u^{1}}{\partial r}+\frac{\partial u^{3}}{\partial x}{>}^{T}\\ \;\;\;\;\;\;\;=[E]\left\{d\right\} \end{array}$$

In this formulation, the column vector {*d*} represents the nodal and modal variables, as defined in Equation (5), while the matrix [*E*] contains the derivatives of the shape functions, defining the strain–displacement relationship within an element. For cylindrical shells composed of linearly cylindrical orthotropic materials, which possess three planes of symmetry aligned with the cylindrical coordinate systems, the orthotropic stress–strain relations for a typical layer are given in Equation (7).

(7) $${\left\{\sigma_{x,\theta ,r}\right\}}_{5\times 1}={\left[D\right]}_{5\times 5}^{l}{\left\{\varepsilon_{x,\theta ,r}\right\}}_{5\times 1}$$

where <*σ*> is the vector, and ${\left[D\right]}_{5\times 5}^{l}$ represents the constitutive matrix for layer *l*, considering the assumption of zero transverse normal stress.

### 2.4. The Formulation of the Element Stiffness Matrix for the Proposed Model

The displacement field {Ω} in an element representing a layer in Equation (5) is expressed in the following form:

{Ω} = [*H*]{*d*} (8)

where [*H*] represents the shape functions associated with the nodal and modal variables {*d*}. The governing equations for a layer are formulated based on the principle of virtual work.

δ*W_ε_* − δ*W_external_* = 0 (9)

where δ*W_ε_* represents the internal virtual strain energy, while δ*W_external_* denotes external virtual work. The internal virtual strain energy within an element of volume *V* can be expressed in terms of the strain vector {*ε*} and the stress vector {*σ*}, as defined in Equations (6) and (7).

(10) $$\delta W_{\varepsilon }\;=\;\int_{V}\delta {\left\{\varepsilon \right\}}^{T}\;\{\sigma \}\;\;dV$$

If the virtual displacements are given by

δ{Ω} = [*H*] δ{*d*} (11)

then the corresponding virtual strain is expressed as follows:

δ{ε} = [*E*] δ{*d*} (12)

The external virtual work induced by the applied load {*S*} on surface *A* is given by

(13) $$\delta W_{external}\;=\;\;\int_{A}\delta {\left\{d\right\}}^{T}\{S\}\;dA$$

Thus, the virtual work equation in Equation (9) is rewritten as follows:

(14) $$\int_{V}\delta {\left\{d\right\}}^{T}\;\;{\left[E\right]}^{T}{\left[D\right]}^{l}\;[E]\;\;\{d\}\;\;dV=\;\;\int_{A}\delta {\left\{d\right\}}^{T}\{S\}dA\;$$

Consequently, the element stiffness matrix of a layer is derived as follows:

(15) $${\left[K\right]}^{l}=\underset{V}{∭}{\left[E\right]}^{T}\;{\left[D\right]}^{l}[E]\;dx\;d\theta \;dr\;$$

**Figure 1:**
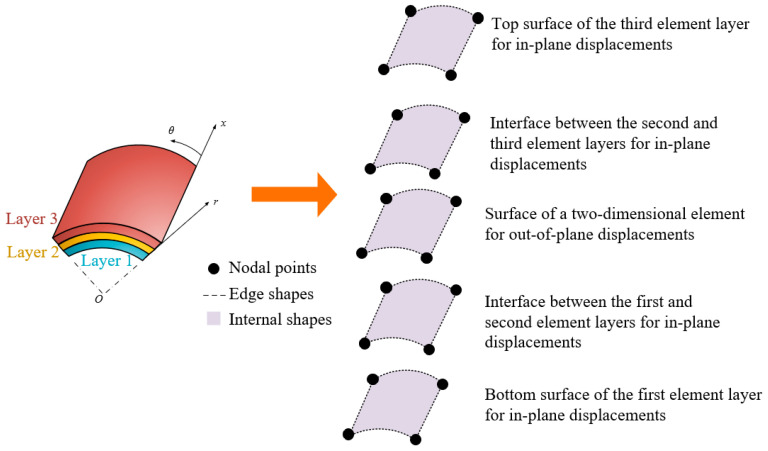
Modeling approach for proposed laminated cylindrical model in *p*-version FEM.

The proposed model is applicable to any number of layers. Figure 1 illustrates its application to a three-layer laminated system. When no gaps or voids are present at the layer interfaces, the compatibility conditions are satisfied, ensuring the continuity of displacement and stress fields. Additionally, as the model is based on LW theory, it allows for modeling gaps and voids, enabling a more accurate representation of the mechanical behavior of various laminated structures. Furthermore, this approach captures both continuity and discontinuity at the interfaces. The in-plane displacement components exhibit a linear variation across the laminate thickness. In contrast, the out-of-plane displacement remains uniform, aligning with the fundamental assumptions of a two-dimensional modeling approach. This facilitates the adoption of the plane stress condition to enhance computational efficiency. This assumption is particularly useful for analyzing thin to moderately thick laminated structures, as it reduces computational complexity while ensuring sufficient numerical accuracy.

### 2.5. VCCT for the Proposed Model for Cylindrical Shells with a Circumferential Crack

The VCCT in linear elastic fracture mechanics is based on the principle that the energy needed to propagate a crack incrementally is identical to the energy required to close it and restore its original state. Instead of explicitly modeling crack propagation, VCCT estimates the strain energy release rate by analyzing the forces and relative displacements at the crack tip. While the conventional VCCT is primarily applied to two-dimensional fracture analysis [35], this study extends its application to a three-dimensional framework for circumferentially cracked cylindrical shells with radius *R*. The proposed approach refines the computation of the total strain energy release rate *G_total_*, across multiple layers, as expressed in the following equation:

(16) $$G_{total}=\frac{F_{x}^{bot}\;\alpha_{x}^{bot}+\;F_{x}^{top}\;\alpha_{x}^{top}+\;F_{\theta }^{bot}\;\alpha_{\theta }^{bot}\;+F_{\theta }^{top}\;\alpha_{\theta }^{top}\;+F_{r}^{bot}\;\alpha_{r}^{bot}+\;F_{r}^{top}\;\alpha_{r}^{top}}{2\;t_{i}\;R\Delta \theta_{a}}$$

**Figure 2:**
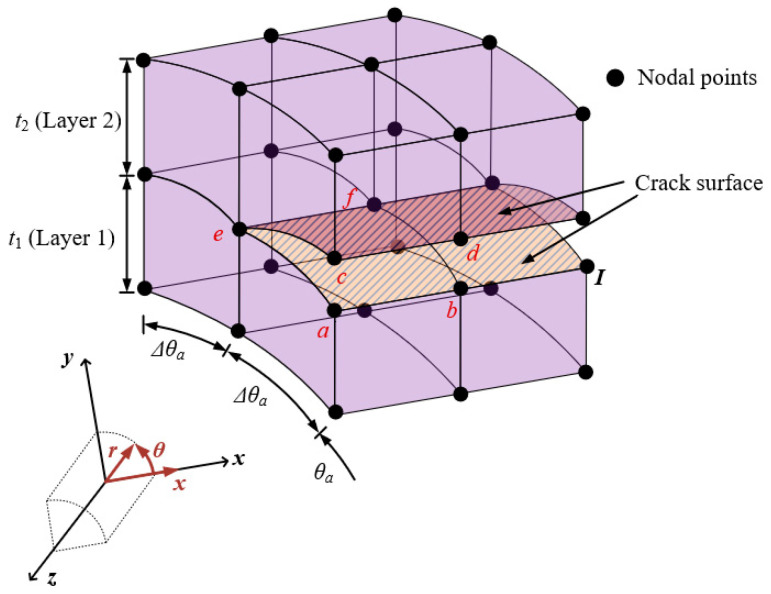
VCCT-based modeling of proposed elements for cylindrical shells with circumferential crack.

Here, the crack tip forces, denoted as *F*, are expressed in cylindrical coordinates. The relative displacements, denoted by *α*, are associated with the nodal variables in the proposed model. The superscripts *bot* and *top* indicate the lower and upper curved surfaces, respectively. Furthermore, the thickness of the *i*th layer is designated as *t_i_*. The relative displacements at each DOF for the nodal points located on the bottom and top surfaces of Layer 1 in Figure 2 can be obtained using the following equation in the cylindrical coordinate system.

(17) $$\alpha_{i}^{bot}\;=\;\alpha_{i}^{a}\;-\alpha_{i}^{b};\;\;\alpha_{i}^{top}\;=\;\alpha_{i}^{c}\;-\;\alpha_{i}^{d}\;\;\;\;\;\;\;\;\;\mathrm{for}\;\;\;\;\;i=x,r,\theta$$

The nodal forces are specified at the nodal points *e* and *f*. Furthermore, the internal nodal forces, denoted as F*, can be obtained from the nodal and modal variables in the proposed elements as follows:

(18) $$F^{*}={\left\{E^{*}\right\}}_{5\times 1}^{T}\;\;{\left[D\right]}_{5\times 5}^{l}\;{\left\{\varepsilon \right\}}_{5\times 1}$$

where the elasticity matrix ${\left[D\right]}_{5\times 5}^{l}$ is introduced in Equation (7), and the strain vector ${\left\{\varepsilon \right\}}_{5\times 1}$ is defined in Equation (6). The strain–displacement vector ${\left\{E^{*}\right\}}_{5\times 1}^{T}$ corresponds to the nodal locations within the strain–displacement matrix [*E*] given in Equation (6). Consequently, the internal force is determined as follows:

(19) $$F^{*}={\left\{E^{*}\right\}}_{5\times 1}^{T}\;\;{\left[D\right]}_{5\times 5}^{l}\;{\left[E\right]}_{5\times n}{\left\{d\right\}}_{n\times 1}$$

where *n* is the total number of degrees of freedom in a single layer.

## 3. Results

### 3.1. Cylindrical Panel with Simply Boundary Condtions

**Figure 3:**
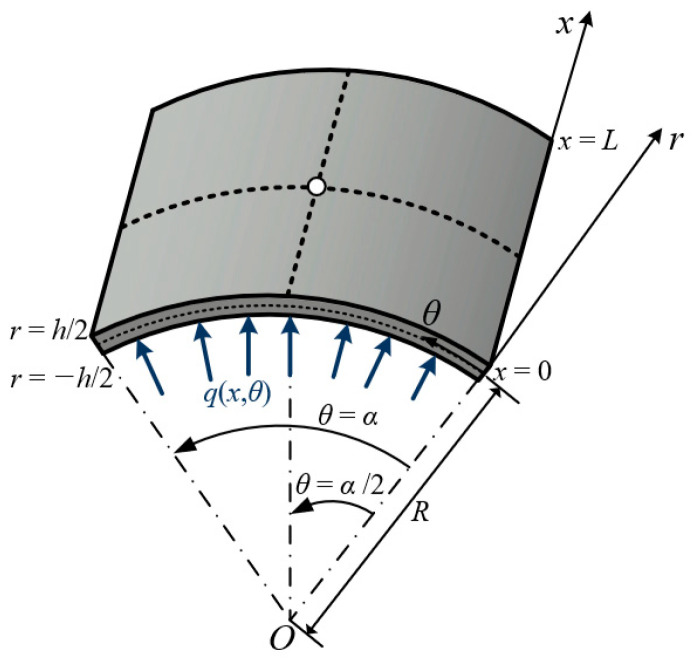
Geometry of simply supported cylindrical panel.

The model proposed in this study was implemented using MATLAB R2023b. Firstly, the structural behavior of cylindrical panels with simple boundary conditions is analyzed. Figure 3 illustrates the panel geometry, which includes a curvature radius of *R* = 1, thickness *h*, and a distributed sinusoidal loading condition *q*, defined as follows:

*q*(*x*,*θ*) = *q*_0_ sin(π*x*/*L*) sin(π*θ*/2*α*) (20)

where *q*_0_ is constant, *α* = π/4 represents the central angle of the panel, and *L* = 4 denotes the span length of the panel.

The geometric parameters and material properties are expressed in dimensionless form. The orthotropic material properties are defined as follows:

*E*_1_ = 25*E*_2_; *G*_12_ = *G*_13_ = 0.5*E*_2_; *G*_23_ = 0.2*E*_2_; *ν*_12_ = 0.25 (21)

The boundary conditions imposed on the panels for displacements *u* are given as follows:

*u_θ_* = 0, *u_r_* = 0 at *x* = 0 and *L*; *u_x_* = 0, *u_r_* = 0 at *θ* = 0 and *α* (22)

Two configurations of laminated cylindrical panels are analyzed, a two-layer (0/90°) configuration and a three-layer (90/0/90°) configuration, where each layer is oriented relative to the *x*-axis. Exploiting symmetry, only a quarter of the panel is considered as the computational domain, with symmetry conditions imposed along the *x*- and *θ*-axes. To verify the proposed model, the displacements *u* and stresses *σ* are considered. The corresponding nondimensionalization equations are presented below.

(23) $$\begin{array}{l} w=u_{r}(L/2,\alpha /2,0)\;\frac{10E_{1}h^{3}}{q_{0}R^{3}};\;\;\;\;\\ \sigma_{x-top}=\sigma_{xx}(L/2,\alpha /2,h/2)\frac{10h^{2}}{q_{0}R^{2}};\;\;\;\sigma_{x-bot}=\sigma_{xx}(L/2,\alpha /2,-h/2)\;\frac{10h^{2}}{q_{0}R^{2}}\\ \sigma_{\theta -top}=\sigma_{\theta \theta }(L/2,\alpha /2,h/2)\;\frac{10h^{2}}{q_{0}R^{2}};\;\;\;\sigma_{\theta -bot}=\sigma_{\theta \theta }(L/2,\alpha /2,-h/2)\;\frac{10h^{2}}{q_{0}R^{2}};\;\;\\ \;\tau_{top}=\sigma_{x\theta }(0,0,h/2)\;\frac{10h^{2}}{q_{0}R^{2}};\;\;\;\;\;\;\;\;\;\;\;\;\;\;\;\;\;\;\;\;\tau_{bot}=\sigma_{x\theta }(0,0,-h/2)\frac{10h^{2}}{q_{0}R^{2}} \end{array}$$

**Figure 4:**
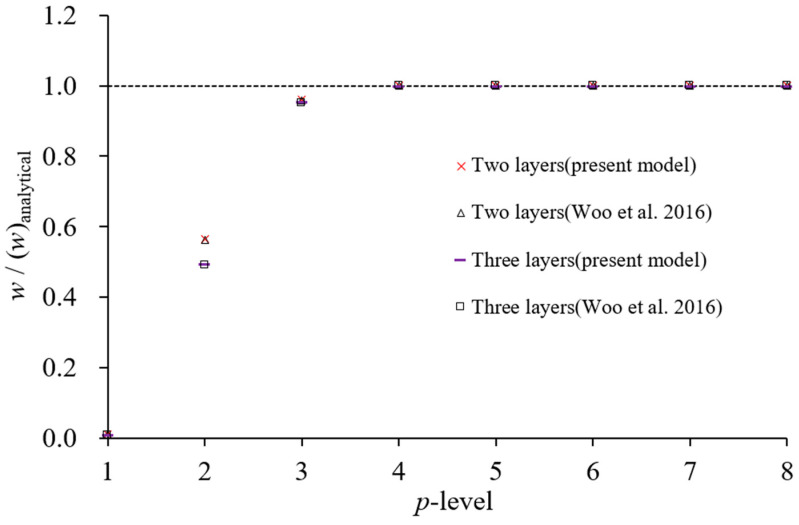
Convergence of maximum deflections values *w* with *p*-refinement [29].

**Figure 5:**
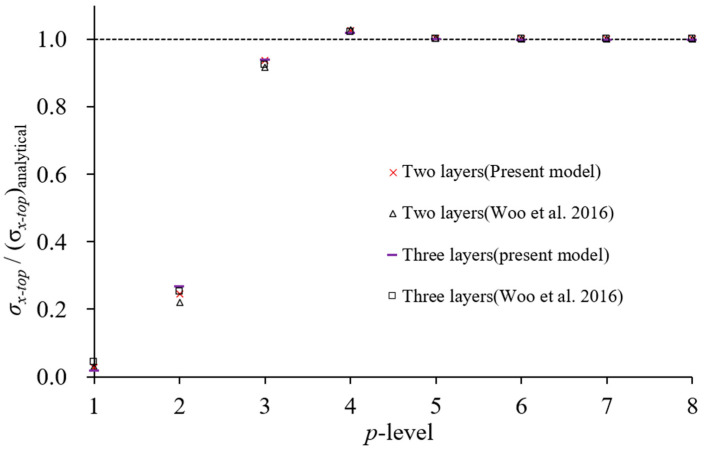
Convergence of stress values *σ_x_* with *p*-refinement [29].

The analysis is conducted using a 2 × 2 mesh for the *xθ* surface. In the thickness direction, two layers are used for the two-layer laminated structure, and three layers are used for the three-layer laminated structure, both along the *r*-coordinate. Figure 4 and Figure 5 illustrate the convergence behavior of displacement and stress with *p*-refinement for a curvature radius-to-thickness ratio of 500. The vertical axis in both figures represents the ratio of computed values to those obtained from the three-dimensional classical analytical method [36]. Reference [29] utilizes an LW model with the same element mesh as in this analysis.

The displacement results converge with negligible differences from the three-dimensional classical analytical values at *p* = 4, while the stress results exhibit close agreement starting from *p* = 5. Similar convergence behavior is observed for other stress parameters. Additional analyses for curvature radius-to-thickness ratios of 100 and 50 confirm the same trend. Table 1 and Table 2 compare the results at *p*-level 6 with those from the existing LW model [29], the three-dimensional classical analysis [36], and the two-dimensional classical analysis [37]. The results based on the LW theory show excellent agreement with the three-dimensional elasticity-based classical analytical solutions. The proposed LW theory-based analysis, formulated on two-dimensional elasticity theory, also exhibits a strong correlation with the three-dimensional classical analysis results. Although some discrepancies appear as the thickness increases, they remain negligible for thin cylindrical shell structures. The proposed analysis model demonstrates nearly identical performance compared to the existing LW theory-based model.

### 3.2. Thin-Walled Cylindrical Shells with Circumferential Cracks

**Figure 6:**
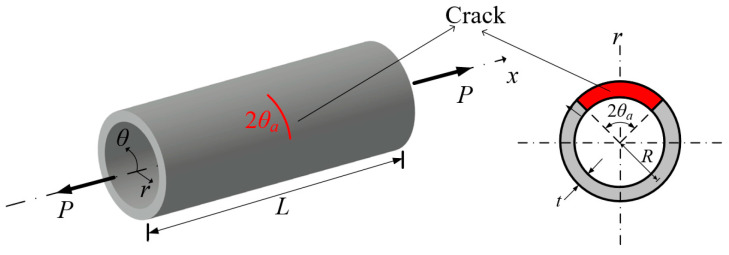
Geometric configuration of cylindrical shell with circumferential crack.

The effectiveness of the proposed method in fracture parameter evaluation is examined through the analysis of unpatched thin-walled cylindrical shells featuring a circumferential crack, as depicted in Figure 6. The considered shell is characterized by a radius *R* of 60 mm, a thickness *t* of 3 mm, and an axial length *L* of 200 mm. The radius-to-thickness ratio of this shell is 20, classifying it as a thin-walled structure. A tensile load *P* of 1 N is applied to the structure, with an elastic modulus *E* of 200 GPa and a Poisson’s ratio of 0.3. The energy release rate *G*, corresponding to the crack angle *θ_a_*, is determined using VCCT. In the opening mode, *G* is nondimensionalized using the following equation [38]:

(24) $$F=\frac{\sqrt{GE}}{\frac{P}{2\pi Rt}\sqrt{\pi R\theta_{a}}}$$

**Figure 7:**
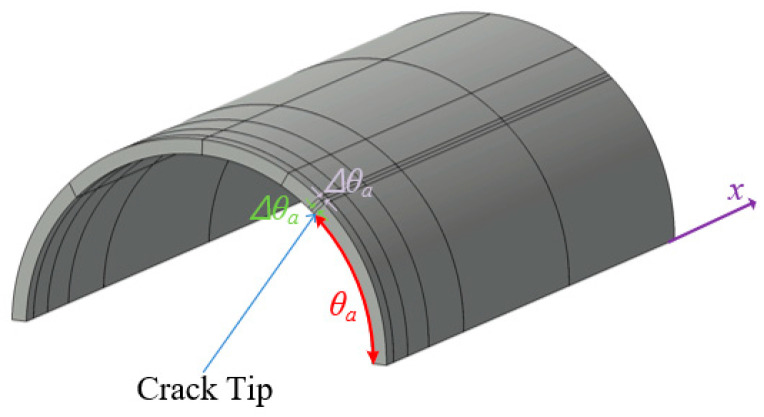
Finite element mesh of proposed model for circumferentially cracked shell without patch.

Leveraging symmetry, the computational domain is restricted to one-quarter of its full extent. The meshing strategy for the computational domain, depicted in Figure 7, follows the proposed modeling framework. A structured mesh consisting of a 6 × 7 element grid is implemented along the *x*- and *θ*-directions, with a single layer of elements spanning the thickness dimension. As illustrated in Figure 7, the meshing approach incorporates a functionally refined structure to enhance computational accuracy.

**Figure 8:**
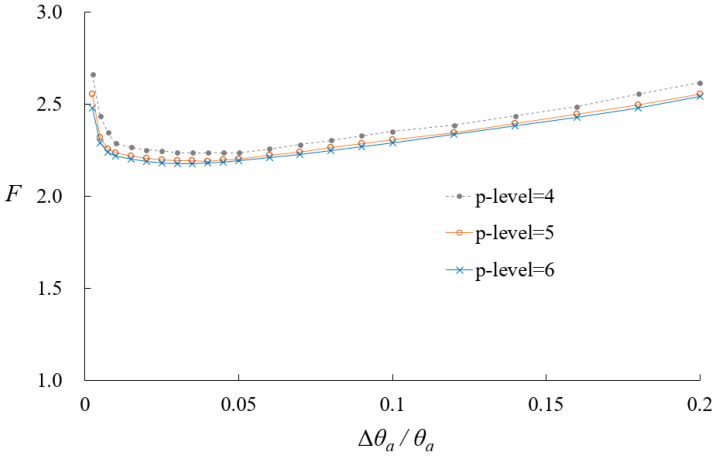
Influence of element size at crack tip on computed nondimensional SIFs.

When applying VCCT to calculate fracture parameters, fine elements of size Δ*θ*_a_ are required near the crack tip. In the case of *h*-refinement, such fine elements result in high aspect ratios, which can degrade element performance in conventional finite element methods. Specifically, *h*-refinement requires an appropriate meshing strategy to address the large aspect ratio problem, necessitating additional mesh refinement according to the size of Δ*θ*_a_. This approach increases computational cost and adds complexity to the modeling process. In contrast, *p*-refinement using Lobatto shape functions effectively addresses these issues. In particular, *p*-level elements of order 5 or higher can maintain accuracy with aspect ratios up to 1000, showing less than 3% relative error [35]. This enables the use of small Δ*θ*_a_ without requiring additional mesh refinement, allowing a relatively coarse mesh to be maintained. Consequently, complex meshing strategies required in *h*-refinement are not needed when using *p*-refinement. In this model, element aspect ratios are kept below 500 to ensure numerical robustness. Additionally, stress oscillations may occur near crack tips when Δ*θ_a_* approaches zero in VCCT [36]. To avoid this, selecting an appropriate Δ*θ_a_* is crucial. Reference [37] suggests that the nondimensional crack extension length should be greater than 0.05 when using conventional finite elements using first- or second-order Lagrangian polynomials. Figure 8 illustrates the effect of Δ*θ*_a_ on VCCT calculations across different *p*-levels when the crack angle is *θ_a_* = 45°. The nondimensional SIFs remain consistent when the nondimensional crack extension length is between 0.01 and 0.05. Table 3 compares the computed nondimensional SIFs for cracked pipes with crack angles from 15° to 75° against existing solutions. The discrepancies are within a 10% relative error, confirming the accuracy and stability of the present model.

### 3.3. Circumferentially Cracked Shells with Composite Patches

**Figure 9:**
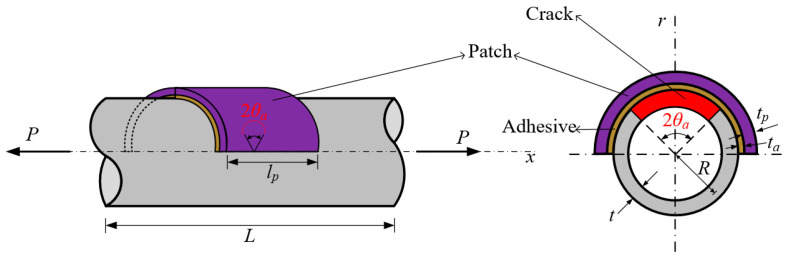
Geometric configuration of circumferentially cracked shell reinforced with composite patch.

Figure 9 illustrates a cylindrical shell with a circumferential crack, reinforced with a composite patch, and subjected to a tensile load *P*. The patch extends 180° around circumference, covering half of the shell’s perimeter, and spans 100 mm along the axial direction. The adhesive layer and patch material have thicknesses of *t_a_* and *t_p_*, respectively. The cylindrical shell maintains the same material properties, geometric dimensions, and applied load as in the previous unpatched case.

**Figure 10:**
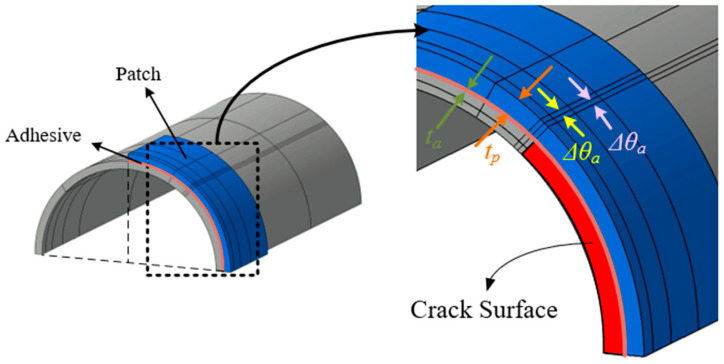
Finite element mesh of circumferentially cracked shell with composite patch.

In this study, composite materials with orthotropic properties, specifically graphite-epoxy and boron-epoxy fiber composites, were considered as patch materials to reinforce cracked structural components. The material properties of these orthotropic patch materials and the adhesive film are presented in Table 4. Figure 10 presents the finite element modeling employed in the current analysis. Similarly to the previous example, the computational domain is reduced to one-quarter of the full structure by considering symmetry. The cracked shell is discretized into a 6 × 7 mesh in the *xθ*-plane, while the patch and adhesive layers are discretized using a 4 × 5 mesh configuration. It is assumed that in the uncracked region, the displacement in the thickness direction is identical across the shell, including the patched region. However, in the cracked region, the displacement in the thickness direction is treated separately for the cracked and patched areas. The previously developed *p*-version LW model [29] can also be modeled using the same mesh configuration, as shown in Figure 10. Consequently, both the proposed model and the LW model contain 82 elements, but there is a difference in the number of DOFs. Table 5 compares the DOF counts for different *p*-levels between the proposed model and the LW model. At the same *p*-level, the proposed model requires approximately one-third of the DOFs needed by the LW model, demonstrating its computational efficiency.

**Figure 11:**
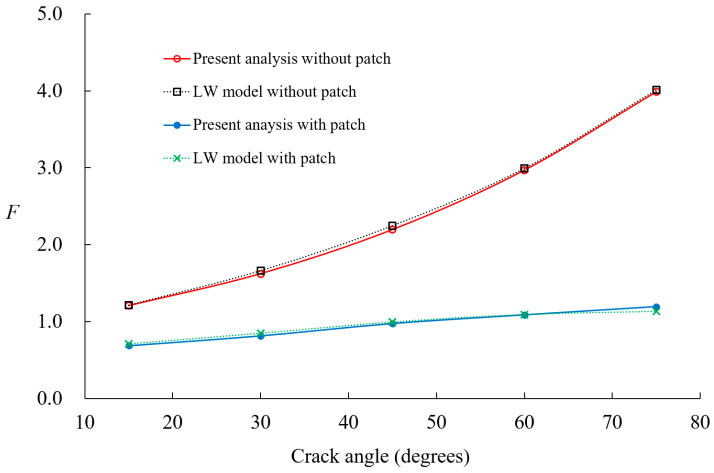
Variations in nondimensional SIFs for circumferentially cracked shells with and without composite patch [29].

Figure 11 illustrates the variation in the normalized SIFs for different crack angles *θ_a_*, in a boron-epoxy patched shell, with patch thickness *t_p_* = 1 mm and adhesive thickness *t_a_* = 0.15 mm. The previously developed *p*-version LW model [18] exhibited behavior nearly identical to that obtained from ANSYS R14.0 simulation results, both before and after patch reinforcement. Figure 11 compares the finite element results for the same *p*-refinement level, confirming that the current analysis model and the previously developed *p*-version LW model show nearly identical behavior. Even with the same coarse mesh, the proposed model achieves higher computational efficiency by requiring significantly fewer DOFs than the previously developed *p*-version LW model. Additionally, as the crack length increases, the difference in SIF values between the unpatched and patched cases becomes more pronounced. This result indicates that the effect of patch reinforcement becomes more significant as the crack size increases, with a particularly pronounced patch effect for crack angles exceeding 45°. Furthermore, as the crack length increases, both patch materials exhibit asymptotic behavior, which is consistent with the findings in reference [41].

**Figure 12:**
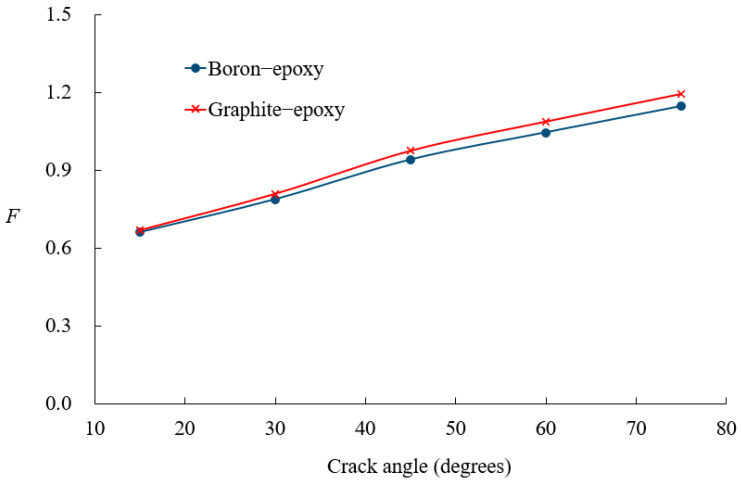
Comparison of nondimensional SIF variations between boron-epoxy and graphite-epoxy patches.

**Figure 13:**
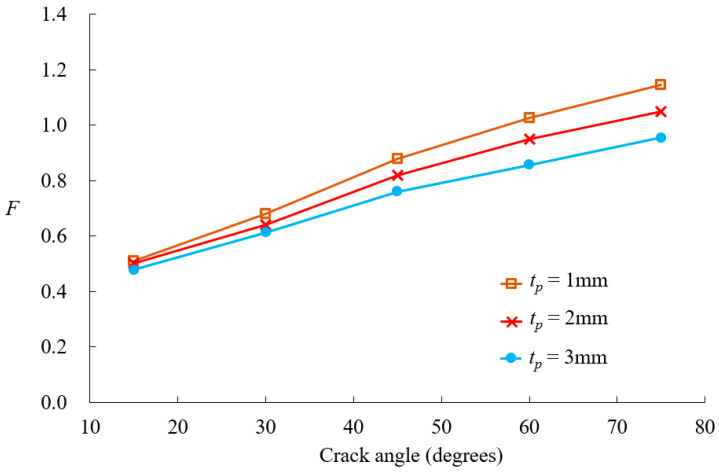
Effect of patch thickness no nondimensional SIFs variation.

Figure 12 illustrates the effect of patch material on crack size behavior, comparing the boron-epoxy and graphite-epoxy while maintaining constant patch dimensions. For small crack sizes, the difference between the two materials is minimal. However, as the crack size increases, the SIF values begin to diverge, suggesting that patch material stiffness plays a more significant role as the crack size grows. Figure 13 investigates the effect of patch thickness on SIF values for the boron-epoxy patch. When the crack size is small, the patch thickness has little impact. However, as the crack size increases, variations in patch thickness lead to noticeable changes in SIF values.

**Figure 14:**
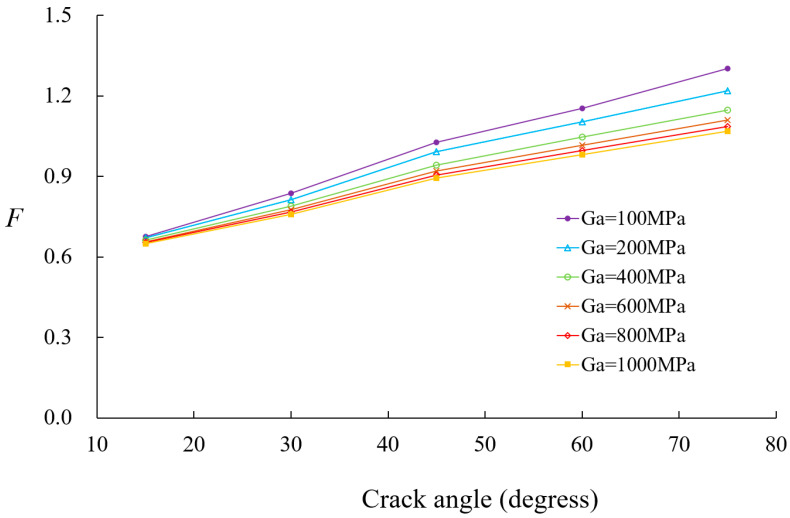
Comparison of adhesive shear modulus on nondimensional SIF variations.

**Figure 15:**
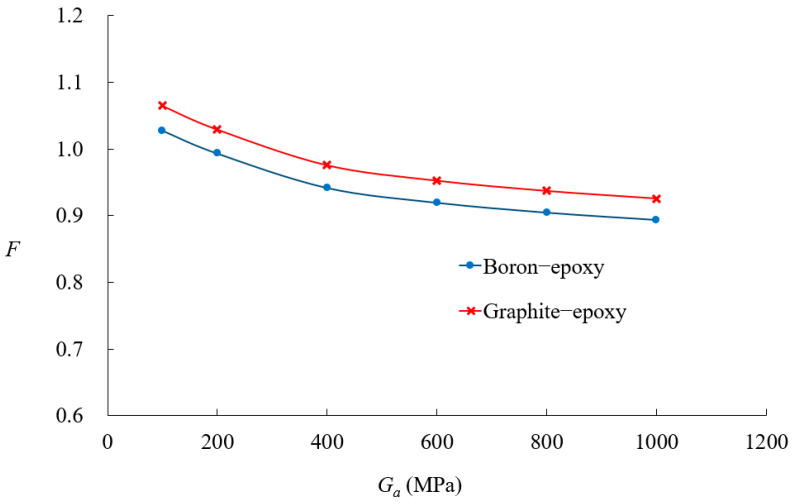
Influence of adhesive shear modulus on nondimensional SIF variations for two patch materials.

**Figure 16:**
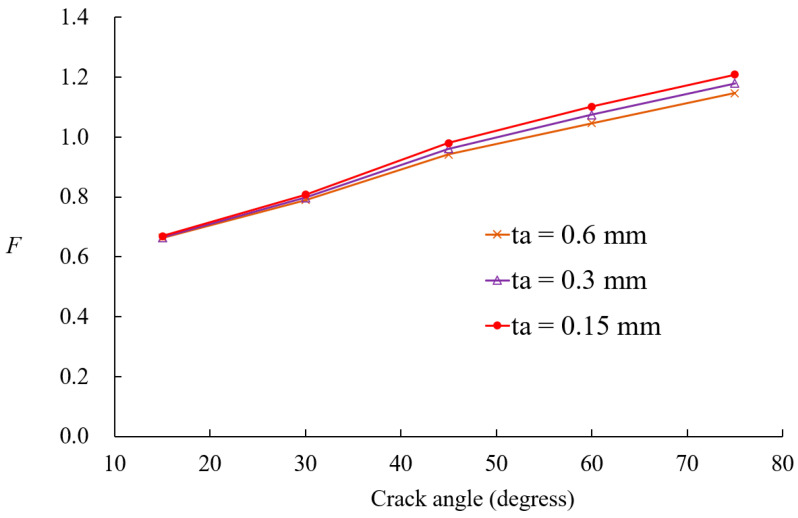
Variations in nondimensional SIFs for different adhesive thicknesses.

Figure 14 presents SIF values for boron-epoxy patching as a function of crack size and adhesive shear modulus. At a crack size of 20°, the adhesive shear modulus has little effect on the SIF values. However, as the crack size increases from 30°, the reduction in SIF values becomes more pronounced with an increase in the adhesive shear modulus. Figure 15 illustrates the variation in SIF values for boron-epoxy and graphite-epoxy patching with respect to changes in adhesive shear modulus, for a crack angle of 45°, with *t_a_* = 0.15 mm. The SIF values decrease as the adhesive shear modulus increases, but beyond 300 MPa, the rate of reduction diminishes significantly. Figure 16 presents the variation in SIF values due to changes in adhesive thickness. Similarly to the previous parameters, patch thickness has a greater influence for crack angles exceeding 45° than for smaller cracks.

## 4. Discussion

The proposed *p*-version finite element model integrating ESL and LW theories demonstrated high computational efficiency and accuracy in analyzing composite-reinforced thin-walled cylindrical shells with circumferential cracks. The results confirm that the model effectively reduces DOFs compared to conventional LW-based models, without compromising numerical accuracy.

The convergence study for cylindrical panels with simple boundary conditions showed that the displacement results converge at *p* = 4, while the stress values stabilize at *p* = 5. These findings indicate that the *p*-version FEM with Lobatto shape functions provides superior numerical stability and reduces the need for excessive mesh refinement. Additionally, the comparison with three-dimensional elasticity solutions and LW theory-based models verified that the proposed model maintains high accuracy, even for relatively thick shell structures.

Fracture analysis using VCCT for unpatched cylindrical shells with circumferential cracks confirmed the model’s ability to predict SIFs accurately. The results were within 10% relative error compared to existing solutions, demonstrating the model’s reliability in fracture mechanics applications. The study also revealed that *h*-refinement increases computational costs, whereas *p*-refinement effectively maintains accuracy without additional mesh refinement, making it a more efficient alternative.

In the case of composite-reinforced cracked shells, the analysis of patch effectiveness indicated that reinforcement becomes more significant as the crack size increases, particularly when the crack angle exceeds 45°. The comparison between the boron-epoxy and graphite-epoxy patches showed that material stiffness has a minor impact on small cracks but becomes increasingly significant for larger cracks. Similarly, the influence of patch thickness and adhesive properties was found to be negligible for small cracks but highly influential for larger cracks.

The study also investigated the role of adhesive shear modulus, revealing that while a higher shear modulus enhances SIF reduction, further increases beyond 300 MPa provide diminishing benefits. This suggests an optimal range for adhesive selection. Thinner adhesives were found to be more effective because thicker adhesives increased bending effects and transferred more load to the patch, thereby reducing reinforcement efficiency.

While the proposed model was validated through numerical comparisons and benchmark studies, the lack of experimental validation remains a limitation of this study. Experimental testing would provide further confirmation of the model’s accuracy and applicability to real-world structural components. Future work should focus on experimental validation of SIF predictions and patch reinforcement effects to enhance the credibility and practical applicability of the proposed approach.

Overall, the findings suggest that *p*-version finite element modeling offers a computationally efficient and accurate approach for analyzing composite-reinforced cylindrical shells with circumferential cracks. The study provides valuable insights into the optimal selection of patch material, thickness, and adhesive properties, which can inform engineering applications in aerospace, marine, and structural rehabilitation.

## 5. Conclusions

The conclusion of this study can be summarized in the following key points:

- The proposed *p*-version finite element model integrating ESL and LW theories significantly improves computational efficiency while maintaining high accuracy in analyzing composite-reinforced cylindrical shells.
- Displacement and stress convergence studies confirmed that the proposed model reduces the need for excessive mesh refinement, achieving accurate results with fewer DOFs compared to conventional LW models.
- The model effectively predicts SIF values for circumferential cracks, with relative errors within 10%, validating its accuracy against existing solutions.
- Composite patch reinforcement effectively reduces SIFs, and its effectiveness for larger cracks, particularly when the crack angle exceeds 45°, is significant.
- Material stiffness has a significant impact on SIFs for large cracks, whereas patch thickness and adhesive properties have minimal influence on small cracks but become critical as crack size increases.
- Optimal adhesive properties were identified, with higher shear modulus improving reinforcement effects, but beyond 300 MPa, further increases provide diminishing benefits. Thinner adhesives were found to be more effective in reducing excessive bending effects.
- The proposed method provides a computationally efficient and robust framework for analyzing composite-reinforced cracked cylindrical shells, with applications in aerospace, marine, and pipeline engineering.

## Figures and Tables

**Table 1:** Deflections and stresses to cylindrical panel with 0/90° laminates.

Variables	*R*/*h*	Present	Reference [18]	Reference [23]	Reference [24]
*w*	50	2.2398	2.2419	2.2420	2.2372
	100	1.3668	1.3669	1.3670	1.3666
	500	0.1005	0.1005	0.1005	0.1005
*σ_x_*_-*top*_	50	0.2188	0.2189	0.2189	0.2187
	100	0.1871	0.1871	0.1871	0.1871
	500	0.0449	0.0449	0.0449	0.0449
*σ_x_*_-*bot*_	50	1.6087	1.6099	1.6100	1.6051
	100	2.2984	2.2998	2.3000	2.2979
	500	0.9436	0.9436	0.9436	0.9436
*σ_θ_*_-*top*_	50	8.9421	8.9368	8.9370	8.9543
	100	5.5630	5.5603	5.5600	5.5643
	500	0.4345	0.4345	0.4345	0.4346
*σ_θ_*_-*bot*_	50	−0.9656	−0.9668	−0.9670	−0.9615
	100	−0.5755	−0.5758	−0.5759	−0.5750
	500	−0.03389	−0.0339	−0.0339	−0.0339
*τ_top_*	50	0.0783	0.0783	0.0784	0.0784
	100	0.1819	0.1819	0.1819	0.1819
	500	0.0925	0.0925	0.0925	0.0925
*τ_bot_*	50	0.3444	0.3444	0.3444	0.3444
	100	0.3414	0.3414	0.3414	0.3414
	500	0.1045	0.1045	0.1045	0.1045

**Table 2:** Deflections and stresses to cylindrical panel with 90/0/90° laminates.

Variables	*R*/*h*	Present	Reference [29]	Reference [36]	Reference [37]
*w*	50	0.5490	0.5494	0.5495	0.5486
	100	0.4712	0.4715	0.4715	0.4711
	500	0.1027	0.1027	0.1027	0.1027
*σ_x_*_-*top*_	50	0.0711	0.0712	0.0712	0.0710
	100	0.0838	0.0838	0.0838	0.0837
	500	0.0559	0.0559	0.0559	0.0559
*σ_x_*_-*bot*_	50	−0.0220	−0.0223	−0.0225	−0.0217
	100	0.0019	0.0018	0.0018	0.0020
	500	0.0379	0.0379	0.0379	0.0379
*σ_θ_*_-*top*_	50	3.9287	3.9299	3.9300	3.9265
	100	3.5068	3.5070	3.5070	3.5048
	500	0.7896	0.7895	0.7895	0.7897
*σ_θ_*_-*bot*_	50	−3.9869	−3.9869	−3.9870	−3.9870
	100	−3.5069	−3.5069	−3.5070	−3.5063
	500	−0.7543	−0.7543	−0.7542	−0.7545
*τ_top_*	50	0.0120	0.0119	0.0118	0.0123
	100	0.0479	0.0478	0.0478	0.0480
	500	0.0766	0.0766	0.0766	0.0766
*τ_bot_*	50	0.0761	0.0760	0.0760	0.0764
	100	0.1038	0.1038	0.1038	0.1039
	500	0.0889	0.0889	0.0889	0.0889

**Table 3:** Comparison of nondimensional SIFs in unpatched shells.

Types	Crack Angles (Degrees)				
	15	30	45	60	75
Present analysis	1.2064	1.6221	2.1954	2.9667	3.9847
Reference [29]	1.2167	1.664	2.2484	2.9913	4.0102
Reference [39]	1.1915	1.5564	2.075	2.8002	3.8295
Reference [40]	1.1854	1.4961	-	-	-

**Table 4:** Materials for composite patch reinforcement (unit: GPa).

Materials	*E*_1_	*E*_2_, *E*_3_	*G*_12_, *G*_13_	*G*_23_	*ν*_12_, *ν*_13_	*ν*_23_
Boron-epoxy	208	25.4	7.24	4.94	0.168	0.035
Graphite-epoxy	172	10.3	4.83	3.10	0.300	0.180
Adhesive	0.965	-	-	-	0.32	-

**Table 5:** Comparison of DOFs between present model and *p*-version LW model.

**Types**	***p*-Level**					
	**2**	**3**	**4**	**5**	**6**	**7**
Present model	1371	2922	5053	7764	11,055	14,926
*p*-version LW model [29]	4122	8760	15,126	23,220	33,042	44,592

## Data Availability

The data presented in this study are available on request from the corresponding author. The data are not publicly available due to privacy.

## Abbreviations

The following abbreviations are used in this manuscript:

DOF	Degrees of freedom
FEM	Finite element method
ESL	Equivalent single-layer
LW	Layer-wise
SIF	Stress intensity factors
VCCT	Virtual crack closure technique
